# Nitrogen Cycling in Soybean Rhizosphere: Sources and Sinks of Nitrous Oxide (N_2_O)

**DOI:** 10.3389/fmicb.2019.01943

**Published:** 2019-08-21

**Authors:** Cristina Sánchez, Kiwamu Minamisawa

**Affiliations:** Graduate School of Life Sciences, Tohoku University, Sendai, Japan

**Keywords:** *Bradyrhizobium*, soybean, rhizosphere, denitrification, N_2_O reductase, *nos* regulation, greenhouse gas, mitigation strategies

## Abstract

Nitrous oxide (N_2_O) is the third most important greenhouse gas after carbon dioxide and methane, and a prominent ozone-depleting substance. Agricultural soils are the primary anthropogenic source of N_2_O because of the constant increase in the use of industrial nitrogen (N) fertilizers. The soybean crop is grown on 6% of the world’s arable land, and its production is expected to increase rapidly in the future. In this review, we summarize the current knowledge on N-cycle in the rhizosphere of soybean plants, particularly sources and sinks of N_2_O. Soybean root nodules are the host of dinitrogen (N_2_)-fixing bacteria from the genus *Bradyrhizobium*. Nodule decomposition is the main source of N_2_O in soybean rhizosphere, where soil organisms mediate the nitrogen transformations that produce N_2_O. This N_2_O is either emitted into the atmosphere or further reduced to N_2_ by the bradyrhizobial N_2_O reductase (N_2_OR), encoded by the *nos* gene cluster. The dominance of *nos*^–^ indigenous populations of soybean bradyrhizobia results in the emission of N_2_O into the atmosphere. Hence, inoculation with *nos*^+^ or *nos*^++^ (mutants with enhanced N_2_OR activity) bradyrhizobia has proved to be promising strategies to reduce N_2_O emission in the field. We discussed these strategies, the molecular mechanisms underlying them, and the future perspectives to develop better options for global mitigation of N_2_O emission from soils.

## Nitrogen Transformations in Soybean Rhizosphere: N_2_O is Emitted Due to Nodule Decomposition

**FIGURE 1:**
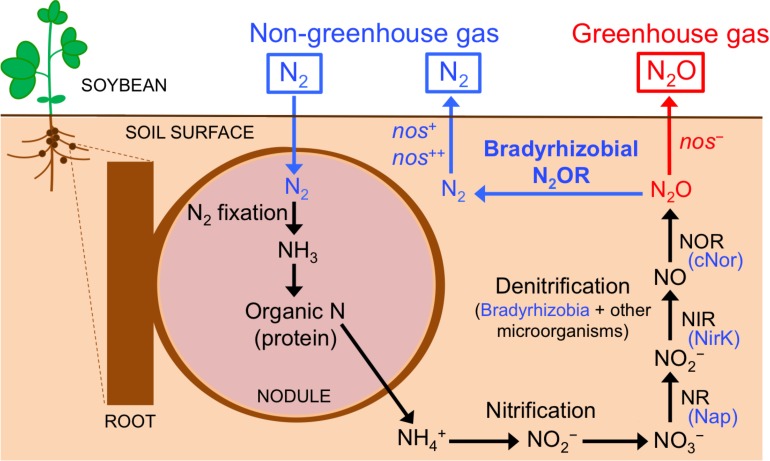
Nitrogen transformations in soybean rhizosphere. Organic nitrogen in the nodule is mineralized into NH_4_^+^ that will be transformed into N_2_O through nitrification and denitrification processes. Soybean bradyrhizobia (blue) and other microorganisms contribute to the denitrification process. The N_2_O formed is either emitted into the atmosphere (red) or further reduced to N_2_ exclusively by soybean bradyrhizobia that produce N_2_O reductase (N_2_OR; blue). Nase, nitrogenase; NR, dissimilatory nitrate reductase; Nap, periplasmic nitrate reductase; NIR, dissimilatory nitrite reductase; NirK, Cu-containing nitrite reductase; NOR, nitric oxide reductase; cNor, *c*-type nitric oxide reductase. See text for more details.

The term, “rhizosphere” was first defined by Lorenz Hiltner as the soil compartment influenced by plant roots. Since then, the rhizosphere has received widespread attention from scientists in different disciplines as a hotspot for intra-microbial and plant-microbe interactions (Hartmann et al., 2008; Bakker et al., 2013). Nitrogen (N), an essential component in living organisms, is the most common limiting nutrient for plant growth in agricultural soils (LeBauer and Treseder, 2008). Fixation of dinitrogen (N_2_) into ammonia (NH_3_) by legume-associated endosymbiotic bacteria, generally known as rhizobia, is a major source of N in soils, and is an agriculturally and ecologically crucial process to reduce plant dependence on industrial N fertilizers. Rhizobia are hosted within the nodules formed in the roots as the result of the symbiosis; inside the nodules, rhizobial cells encounter oxygen-limiting conditions that are required for the synthesis and activity of nitrogenase that converts N_2_ into NH_3_ (Figure 1). Thus, rhizobia provide N to the host plant, which in return, supplies photosynthetically fixed carbon to the bacteria. Although rhizobia have been extensively studied as inhabitants of legume nodules, only few studies have focused on them as denitrifiers in the legume rhizosphere (Inaba et al., 2009, 2012; Shiina et al., 2014; Saeki et al., 2017).

Soybean [*Glycine max* (L.) Merr.] is grown on 6% of the world’s arable land. Its production increased from 17 to 230 million metric tons in the past 50 years, and is expected to increase rapidly in the future due to an increased demand for soybean meal and oil (Uchida and Akiyama, 2013). Soybean generally hosts rhizobia from the genus *Bradyrhizobium* (Argaw, 2014). In addition to fixing N_2_, many soybean-associated *Bradyrhizobium* strains contain genes for some, or all of the four denitrification reductases. Denitrification is an alternate respiratory process in which the oxidized forms of N in the soil – nitrate (NO_3_^–^) and nitrite (NO_2_^–^) – are used as electron acceptors in oxygen limiting conditions. NO_2_^–^ is reduced to nitric oxide (NO), nitrous oxide (N_2_O), and N_2_ gases, which are returned to the atmosphere (Figure 1). The complete denitrification pathway in soybean bradyrhizobia requires four enzymes, periplasmic NO_3_^–^ reductase (Nap), copper (Cu)-containing NO_2_^–^ reductase (NirK), *c*-type NO reductase (cNor), and N_2_O reductase (N_2_OR) (Figure 1). Bradyrhizobial denitrification is functional under both free-living (for example, in the soybean rhizosphere) and symbiotic (inside the root nodules) conditions (Sameshima-Saito et al., 2006a; Sánchez et al., 2011; Inaba et al., 2012).

N_2_O is the third most important greenhouse gas after carbon dioxide (CO_2_) and methane (CH_4_) and is currently the major ozone-depleting compound in the stratosphere (Hénault et al., 2012; Thomson et al., 2012). Terrestrial ecosystems are the main source of N_2_O, primarily due to the use of industrial N fertilizers in agriculture (Hénault et al., 2012; Thomson et al., 2012; Intergovernmental Panel on Climate Change [IPCC], 2014). The soybean rhizosphere is a hotspot for N transformations including production and removal of N_2_O. Nodule decomposition is a major source of N_2_O, particularly in soybean ecosystems, compared to other possible sources including aboveground plant residues. Inaba et al. (2009) showed that N_2_O is only emitted by decomposed nodules, but not by fresh nodules or roots. Studies showed that N_2_O emission not only occurs from decomposed nodules after soybean harvesting, but also starts before the harvest till the late growth period (Yang and Cai, 2005; Inaba et al., 2012; reviewed by Uchida and Akiyama, 2013). Biological N_2_ fixation is an important indirect source for N_2_O during nodule decomposition. Indeed, a ^15^N tracer experiment revealed that the N_2_O emitted from the soybean rhizosphere was almost entirely derived from N_2_ fixed symbiotically in the nodules (Inaba et al., 2012; Figure 1). During nodule decomposition, rhizospheric microbes are essential for N_2_O emission; organic N from the nodule is mineralized into ammonium (NH_4_^+^, Figure 1); N_2_O is then produced via nitrification and denitrification (Inaba et al., 2009, 2012; Figure 1). Although soybean bradyrhizobia are important players in denitrification (responsible for ∼41% of the total N_2_O produced), but other denitrifying microorganisms are also important contributors (∼59% of the total N_2_O produced; Inaba et al., 2012). Populations of nematodes, protozoans, and fungi were markedly enhanced in the soybean rhizosphere of decomposing nodules, suggesting that these organisms contributed to the complex N transformation (Inaba et al., 2009). N_2_O formed by denitrification is either emitted into the atmosphere or is further reduced to N_2_ by N_2_O reductase of soybean bradyrhizobia (Sameshima-Saito et al., 2004, 2006b; Inaba et al., 2012; Figure 1). In soybean fields, both N_2_- and N_2_O-producing soybean bradyrhizobia strains coexist; therefore soybean roots are infected with multiple bradyrhizobial strains that differ in denitrifying activity (Sameshima-Saito et al., 2004, 2006b; Shiina et al., 2014). Thus, the flux of N_2_O from soybean fields during nodule decomposition is partly determined by biotic factors like the balance between N_2_O emission by soil microbes whose denitrification produce N_2_O (including bradyrhizobia) and N_2_O uptake by soybean bradyrhizobia that produce N_2_OR (Inaba et al., 2012; Figure 1).

## Nitrous Oxide Reductase: The Key Enzyme to Reduce N_2_O Emission

N_2_OR is a Cu-containing enzyme that catalyzes the two-electron reduction of N_2_O to N_2_, which is the only known pathway for the removal of N_2_O from ecosystems (Richardson et al., 2009). Therefore, the expression and activity of N_2_OR is a natural target to mitigate N_2_O emission from agricultural soils.

In *Bradyrhizobium diazoefficiens* (reclassified from *Bradyrhizobium japonicum* by Delamuta et al., 2013), the N_2_OR (NosZ) and its accessory functions are encoded by *nosRZDYFLX* gene cluster (Velasco et al., 2004). The flavoproteins NosR and NosX form an electron transport pathway from the quinone pool to NosZ; NosR is also required for the transcription of *nos* genes (Velasco et al., 2004; Zumft and Kroneck, 2007). NosD, NosF, NosY, and NosL are involved in maturation of the Cu_*Z*_ site of NosZ (Zumft and Kroneck, 2007). Although the reduction of N_2_O to N_2_ by N_2_OR is integrated as the last step of the denitrification pathway, it can provide a benefit for N_2_O respiration as a separate module. When N_2_O is provided as the sole electron acceptor to *B. diazoefficiens*, anaerobic respiration and growth are sustained by reducing N_2_O to N_2_ (Zumft, 1997; Sánchez et al., 2013; Graf et al., 2014).

*Bradyrhizobium diazoefficiens* carries the *nos* gene cluster (*nos*^+^) and denitrifies NO_3_^–^ to N_2_, whereas other soybean bradyrhizobia including *B. japonicum* lack the *nos* gene cluster (*nos*^–^) and cannot reduce N_2_O to N_2_ (Sameshima-Saito et al., 2004, 2006b; Inaba et al., 2012). Sameshima-Saito et al. (2006a) showed that soybean roots nodulated with *B. diazoefficiens* could scavenge very low concentrations of exogenous N_2_O, equivalent to the natural concentration of N_2_O in air (∼0.34 ppm; Badr and Probert, 1992). Later, pot studies demonstrated that soybean roots inoculated with *nos*^+^ strains have the potential to reduce N_2_O derived from decomposing nodules and other N sources from fertilizer and soil organic matter (Hénault and Revellin, 2011; Inaba et al., 2012; Uchida and Akiyama, 2013). Thus, *Bradyrhizobium nos*^+^ strain inoculation is a promising strategy for mitigating N_2_O emission at the field scale. This is likely effective in soybean soils that act as an N_2_O source, a condition that potentially arises from several situations like (i) indigenous bradyrhizobia community being dominated by *nos*^–^ species (Sameshima-Saito et al., 2004, 2006b; Shiina et al., 2014), (ii) anoxic conditions such as waterlogging that induce N_2_O emissions from denitrification by *Bradyrhizobium* (Tortosa et al., 2016) and other microorganisms, and (iii) increased NO_3_^–^ supply as a consequence of heavy N fertilization leading to increased N_2_O emission from intact soybean root systems via bradyrhizobial denitrification (Ciampitti et al., 2008; Hirayama et al., 2011; Inaba et al., 2012).

Among the N_2_O-mitigation options for agricultural soils, the first biological method for the field scale was described by Itakura et al. (2013). Mutants of *B. diazoefficiens* USDA110 with a high *nos* expression and N_2_OR activity (*nos*^++^ strains) were generated by a mutational strategy (Itakura et al., 2008). This strategy involved (1) introduction of a plasmid containing a mutated *dnaQ* gene (pKQ2) to enhance replication error on the *B. diazoefficiens* genome by disrupting the exonuclease proofreading activity of DNA polymerase, (2) enrichment culture under selection pressure favoring anaerobic N_2_O respiration, and (3) elimination of the pKQ2 plasmid by nodulation. Thus, the resulting mutants were not genetically modified organisms (GMOs). The *nos*^++^ mutants retained higher *nos* expression and N_2_OR activity in both free-living and symbiotic cells than the wild-type *nos*^+^ strains (Itakura et al., 2013; Sánchez et al., 2014). Comparative analysis of the *nos*^++^ mutant genomes revealed the mechanism underlying the *nos*^++^ phenotype, a point mutation in *nasS* gene encoding the NO_3_^–^ sensor of the two-component NasST regulatory system (Sánchez et al., 2014, 2017), which will be discussed later.

The effectiveness of N_2_O emission mitigation by the *nos*^++^ mutant was first confirmed under simulated field conditions in a pot experiment with Andosol soil, which predominantly contains *nos*^–^ bradyrhizobia (Itakura et al., 2013). N_2_O emission from the Andosol soil inoculated with the *nos*^++^ mutant strain was significantly reduced compared with that inoculated with wild-type *nos*^+^ strain. Itakura et al. (2013) demonstrated that inoculation of *nos*^++^ strains to growing soybean in Andosol soil reduced postharvest N_2_O emission by 43% in the lysimeter study and by 54% in the farm-scale study. However, reduction in postharvest N_2_O emission by inoculation with the *nos*^++^ strains was not significant in a Gleysol soil, which predominantly contains *nos*^+^ bradyrhizobia, although the *nos*^++^ strains clearly showed higher N_2_O-reducing potential than that of the *nos*^+^ strains under laboratory conditions (Itakura et al., 2013; Shiina et al., 2014). Thus, some factors present in the soybean rhizosphere of Gleysol soil limited the potential N_2_O mitigation ability of the *nos*^++^ strains.

A recent report showed that inoculation of soil with a mixed and enriched culture of indigenous *nos*^+^ strains of the *B. diazoefficiens* USDA110 group isolated from agricultural fields efficiently mitigated N_2_O emission (Akiyama et al., 2016). As in the *nos*^++^ approach above, inoculation with the mixed culture was successful in soils dominated by *nos*^–^ bradyrhizobia. Additionally, this mixture is expected to be more competitive and adaptable to changing environmental factors than a single strain (Akiyama et al., 2016). This method is an alternative to GMOs and overcomes the problem of strong opposition to them.

## Regulation of N_2_O Reductase Genes in Bradyrhizobia

**FIGURE 2:**
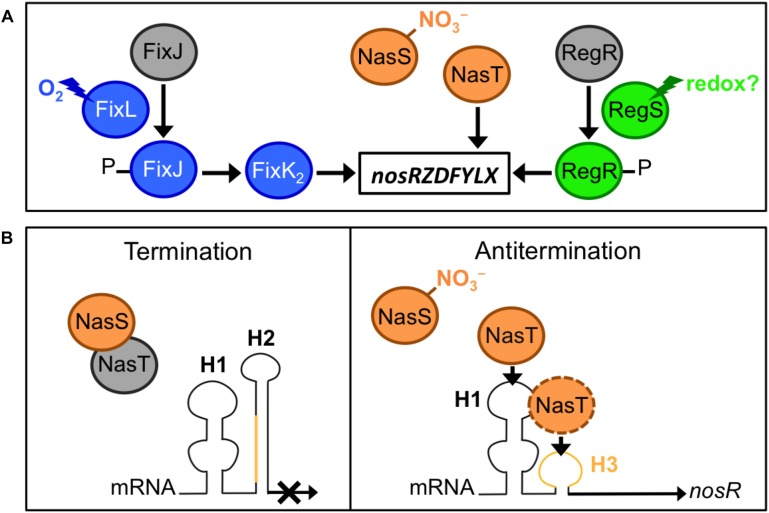
Regulation of N_2_O reductase genes in bradyrhizobia. **(A)** Environmental factors and regulatory proteins involved in the control of *nos* expression. **(B)** The mechanism for NasT-mediated transcriptional antitermination of the *nos* genes. In the absence of nitrate (NO_3_^–^), NasT is sequestered by NasS; thus in the absence of NasT, the native conformation of the *nosR*-leader mRNA, which contains the H1 and H2 hairpins, is responsible for the termination of the *nos* transcription. When a certain level of NO_3_^–^ is sensed by NasS, NasT is dissociated from NasS–NasT complex; the binding of NasT to H1 (and likely to H3 region, in orange) results in a conformational change in the mRNA that allows the read-through transcription of *nos* genes.

Considering the importance of N_2_OR in N_2_O removal from ecosystems, significant progress has been made in understanding its genetic regulation in bacteria, especially in the denitrifying bacteria, *Paracoccus denitrificans* (reviewed by Gaimster et al., 2018), and *B. diazoefficiens* as a model for denitrification in legume-associated rhizobia. In the latter bacterium, the *nosR* gene is constitutively expressed at a low level from the promoter P_*a*_ in aerobiosis, but is strongly induced from the promoter P_*d*_ under denitrifying conditions (i.e., anoxia with NO_3_^–^ as electron acceptor), which is dependent on the oxygen-responsive regulatory cascade FixLJ–FixK_2_ (Torres et al., 2016, 2017; Sánchez et al., 2017; Figure 2A). Decreasing oxygen level to 5% during a culture triggers ATP-dependent autophosphorylation of the heme-based sensor kinase, FixL to phosphorylate the response regulator FixJ, which activates FixK_2_, a transcriptional activator that directly interacts with the *nosR* promoter (Torres et al., 2016, 2017; Figure 2A). Although the FixLJ–FixK_2_ cascade has been considered as the main regulator for *nos* genes in bradyrhizobia for a long time, the NasST two-component system has been revealed as an important regulator of *nos* transcription in response to NO_3_^–^ under both aerobic and anaerobic conditions (Sánchez et al., 2014, 2017; Figure 2A). The *nasST* operon encodes a NO_3_^–^ and NO_2_^–^ sensor/transcriptional antitermination regulatory system. This system was initially considered to be involved in the NO_3_^–^/NO_2_^–^-responsive regulation of the *nas* genes for the NO_3_^–^ assimilation pathway in bacteria, including *B. diazoefficiens* (Romeo et al., 2012; Wang et al., 2012; Luque-Almagro et al., 2013; Cabrera et al., 2016). A recent transcriptomic study using RNA-seq has shown that most of the genes whose expression changed in the *B. diazoefficiens* Δ*nasT* mutant are related to N metabolism, especially amino acid transport (Sánchez et al., 2019).

NasS contains a NO_3_^–^/NO_2_^–^-binding motif similar to that of NrtA, which is the periplasmic component of an ABC-type system for NO_3_^–^ and NO_2_^–^ uptake in cyanobacteria (Koropatkin et al., 2006). NasT is an ANTAR (AmiR and NasR transcription antitermination regulator)-family protein (Shu and Zhulin, 2002). NasS and NasT form a complex that dissociates when NasS senses NO_3_^–^ in micromolar concentrations (Luque-Almagro et al., 2013; Sánchez et al., 2014; Hidaka et al., 2016). When NO_3_^–^ is present, *nos* expression is markedly decreased (∼70%) in the Δ*nasT* background. In absence of NO_3_^–^, *nos* expression is induced in the Δ*nasS* background but such induction is abolished with the additional deletion of *nasT*. Thus, NO_3_^–^ counteracts the NasS-mediated inhibition of *nos* by allowing the dissociation of the antiterminator NasT from the NasS-NasT complex (Sánchez et al., 2014, 2017; Figure 2). Then, the application of *nos*^++^ mutants (carrying a mutation in *nasS*) may be more effective than that of wild type *nos*^+^ alone if NO_3_^–^ concentration in the rhizosphere is below the threshold for dissociation of the NasS-NasT complex. Although the concentration threshold *in vitro* is within the micromolar range (Hidaka et al., 2016), this concentration remains to be fixed under soil conditions.

When NasT is released from NasS, NasT interacts directly with a 5′-leader region of the *nosR* mRNA and interferes with the formation of a terminator structure, allowing a read-through transcription of *nos* genes (Sánchez et al., 2017; Figure 2B). The transcription terminator located upstream of *nosR* comprises two RNA-hairpin structures (H1 and H2); the binding of NasT to H1 induces a conformational change in the terminator and facilitates read-through transcription to induce *nos* expression (Sánchez et al., 2017; Figure 2B). Deletion of either H1 or H2 increases *nos* expression in the absence or presence of NO_3_^–^ (Sánchez et al., 2014, 2017; Figure 2). Thus, theoretically, a *B. diazoefficiens* mutant defective in H1 (Figure 2B) would be an ideal *nos*^++^ inoculant, because (i) it is expected to specifically induce *nos* genes, whereas *nos*^++^ strains derived from *nasS* mutations affected other genes controlled by the NasST system, and (ii) *nos* induction is independent of soil NO_3_^–^ concentration (Itakura et al., 2013; Sánchez et al., 2017, 2019). Mutation of H1 may be applicable to other agriculturally important *nos*^+^ bacteria such as *Bradyrhizobium oligotrophicum* S58, an endophyte of rice roots – where it potentially fixes N_2_ (Ohta and Hattori, 1983; Okubo et al., 2013; Sánchez et al., 2017; Sánchez and Minamisawa, 2018).

Furthermore, studies on *P. denitrificans* have shown that reduction of N_2_O to N_2_ is dependent on Cu, a key cofactor of the Nos enzyme. Thus, bacterial cultures lacking Cu accumulate significant amounts of N_2_O (Felgate et al., 2012). Cu deficiency results in a decreased expression of *nosZ* (Sullivan et al., 2013). Another key factor is the pH that significantly affects N_2_O emission from microbes. The expression of functional N_2_OR is difficult at low pH (Bakken et al., 2012). *Sinorhizobium meliloti*, the alfalfa endosymbiont, is unable to express N_2_OR at pH 6 (Bueno et al., 2015). In *P. denitrificans*, pH has little effect on the transcription of the *nosZ*, but may have a direct posttranslational effect on the assembly and/or activity of the N_2_OR holoenzyme (Bakken et al., 2012). The effect of Cu or pH on the reduction of N_2_O to N_2_ in *B. diazoefficiens* is currently unknown. Among the environmental factors that affect the bacterial N_2_OR activity, very little is known about the effect of availability and redox state of carbon sources. In this context, the response regulator RegR of the RegSR two-component regulatory system appears to induce *nos* expression in *B. diazoefficiens*, most likely in response to redox state (Torres et al., 2016; Figure 2A).

## Future Directions for Production of Bradyrhizobial Inoculants for N_2_O Mitigation

The understanding of N_2_O production in the soybean rhizosphere has been significantly advanced. A variety of techniques, such as functional omics, ^15^N isotope analysis, and zymography, will facilitate a better understanding of the players and processes for N transformation in the soybean rhizosphere of degrading nodules. In addition, further studies on soil factors that control the amount and distribution of soybean bradyrhizobia in the rhizosphere are required because they are key determinants for the flux of N_2_O during nodule decomposition (Inaba et al., 2012).

Shiina et al. (2014) reported that the soil type determines the occurrence of *B. diazoefficiens* (*nos*^+^) or *B. japonicum* (*nos*^–^) in Japanese soybean fields; the *nos*^+^ bradyrhizobia are predominant in Gleysol (wetland soils where water regime causes low-oxygen conditions), whereas the *nos*^–^ bradyrhizobia are predominant in Andosols (volcanic soils containing porous sediments, resulting in more aerated conditions). Saeki et al. (2017) reported that the presence of *nos* in *B. diazoefficiens* confers a competitive advantage in flooded soils with low-oxygen conditions, similar to Gleysol soils. However, batch experiments suggested that *B. japonicum* may be less competitive compared to *B. diazoefficiens* due to energy depletion under anaerobic conditions, which is associated with a marked impairment of Nap activity in *B. japonicum* and not with the absence of *nos* (Siqueira et al., 2017). These findings emphasize the need for further research on how soil factors influence the relevance of the N_2_O reduction step in bradyrhizobial competition.

Significant advances have led to the use of bradyrhizobial N_2_OR as an N_2_O sink in soybean ecosystems. Following the work done by Itakura et al. (2013) and Akiyama et al. (2016), promising strategies for production of rhizobial inoculants for N_2_O mitigation would be the selection of superior native strains (in terms of adaptation to local environments and N_2_-fixing symbiotic efficiency) and the optimization of N_2_O reduction activity through appropriate genetic modification or management of soil chemical and physical properties. However, generating mutants requires more time, cost, and technical skill than isolating *nos*^+^ strains from local soybean fields (Itakura et al., 2013; Akiyama et al., 2016). Moreover, inoculating a mixture of native strains provides more adaptability than a single strain (Akiyama et al., 2016). Thus, isolating *nos*^+^ strains from local fields is more feasible for many soybean-producing countries and is potentially applicable to other ecosystems. Indeed, it has already been suggested the potential activity of *Ensifer* (formerly *Sinorhizobium*) *meliloti*, the alfalfa endosymbiont (Bueno et al., 2015).

## Author Contributions

Both authors have contributed equally to the discussion, writing, and approving the manuscript.

## Conflict of Interest Statement

The authors declare that the research was conducted in the absence of any commercial or financial relationships that could be construed as a potential conflict of interest.

## References

[B1] Akiyama H. Hoshino Y. T. Itakura M. Shimomura Y. Wang Y. Yamamoto A. (2016). Mitigation of soil N2O emission by inoculation with a mixed culture of indigenous *Bradyrhizobium diazoefficiens*. *Sci. Rep.* 6:32869. 10.1038/srep3286910.1038/srep32869 27633524 PMC5025649

[B2] Argaw A. (2014). Symbiotic effectiveness of inoculation with *Bradyrhizobium* isolates on soybean [*Glycine max* (L.) *Merrill]* genotypes with different maturities. *Springer Plus.* 3:753. 10.1186/2193-1801-3-753 26034703 PMC4447725

[B3] Badr O. Probert S. D. (1992). Nitrous oxide in the Earth’s atmosphere. *Appl. Energ.* 41 177–200. 10.1016/0306-2619(92)90002-s

[B4] Bakken L. R. Bergaust L. Liu B. Frostegard Å (2012). Regulation of denitrification at the cellular level: a clue to the understanding of N2O emissions from soils Phil. *Trans. R. Soc. B.* 367 1224–1234. 10.1098/rstb.2011.0321 22451108 PMC3306626

[B5] Bakker P. A. Berendsen R. L. Doornbos R. F. Wintermans P. C. Pieterse C. M. (2013). The rhizosphere revisited: root microbiomics. *Front. Plant Sci.* 4:165. 10.3389/fpls.2013.00165 23755059 PMC3667247

[B6] Bueno E. Mania D. Frostegard Å Bedmar E. J. Bakken L. R. Delgado M. J. (2015). Anoxic growth of *Ensifer meliloti* 1021 by N2O-reduction, a potential mitigation strategy. *Front. Microbiol.* 6:537. 10.3389/fmicb.2015.00537 26074913 PMC4443521

[B7] Cabrera J. J. Salas A. Torres M. J. Bedmar E. J. Richardson D. J. Gates A. J. (2016). An integrated biochemical system for nitrate assimilation and nitric oxide detoxification in *Bradyrhizobium japonicum*. *Biochem. J.* 473 297–309. 10.1042/BJ20150880 26564204 PMC4724949

[B8] Ciampitti I. A. Ciarlo E. A. Conti M. E. (2008). Nitrous oxide emissions from soil during soybean [(*Glycine max* (L.) *Merrill*] crop phenological stages and stubbles decomposition period. *Biol. Fertil. Soils* 44 581–588. 10.1007/s00374-007-0241-7

[B9] Delamuta J. R. Ribeiro R. A. OrmeñÞo-Orrillo E. Melo I. S. Martínez-Romero E. Hungria M. (2013). Polyphasic evidence supporting the reclassification of *Bradyrhizobium japonicum* group Ia strains as *Bradyrhizobium diazoefficiens* sp. nov. *Int. J. Syst. Evol. Microbiol.* 63 3342–3351. 10.1099/ijs.0.049130-0 23504968

[B10] Felgate H. Giannopoulos G. Sullivan M. J. Gates A. J. Clarke T. A. Baggs E. (2012). The impact of copper, nitrate and carbon status on the emission of nitrous oxide by two species of bacteria with biochemically distinct denitrification pathways. *Environ. Microbiol.* 14 1788–1800. 10.1111/j.1462-2920.2012.02789.x 22642644

[B11] Gaimster H. Alston M. Richardson D. J. Gates A. J. Rowley G. (2018). Transcriptional and environmental control of bacterial denitrification and N2O emissions. *FEMS Microbiol. Lett.* 365:fnx277. 10.1093/femsle/fnx277 29272423

[B12] Graf D. R. Jones C. M. Hallin S. (2014). Intergenomic comparisons highlight modularity of the denitrification pathway and underpin the importance of community structure for N2O emissions. *PLoS One* 9:e114118. 10.1371/journal.pone.0114118 25436772 PMC4250227

[B13] Hartmann A. Rothballer S. Schmid M. (2008). Lorenz Hiltner, a pioneer in rhizosphere microbial ecology and soil bacteriology research. *Plant Soil* 312 7–14. 10.1007/s11104-007-9514-z

[B14] Hénault C. Grossel A. Mary B. Roussel M. Leonard J. (2012). Nitrous oxide emission by agricultural soils: a review of spatial and temporal variability for mitigation. *Pedosphere* 22 426–433. 10.1016/s1002-0160(12)60029-0

[B15] Hénault C. Revellin C. (2011). Inoculants of leguminous crops for mitigating soil emissions of the greenhouse gas nitrous oxide. *Plant Soil* 346 289–296. 10.1007/s11104-011-0820-0

[B16] Hidaka M. Gotoh A. Shimizu T. Minamisawa K. Imamura H. Uchida T. (2016). Visualization of NO3-/NO2- dynamics in living cells by fluorescence resonance energy transfer (FRET) imaging employing a rhizobial two-component regulatory system. *J. Biol. Chem.* 291 2260–2269. 10.1074/jbc.M115.687632 26631727 PMC4732209

[B17] Hirayama J. Eda S. Mitsui H. Minamisawa K. (2011). Nitrate-dependent N2O emission from intact soybean nodules via denitrification by *Bradyrhizobium japonicum* bacteroids. *Appl. Environ. Microbiol.* 77 8787–8790. 10.1128/aem.06262-11 22003029 PMC3233077

[B18] Inaba S. Ikenishi F. Itakura M. Kikuchi M. Eda S. Chiba N. (2012). N2O emission from degraded soybean nodules depends on denitrification by *Bradyrhizobium japonicum* and other microbes in the rhizosphere. *Microbes Environ.* 27 470–476. 10.1264/jsme2.me12100 23047151 PMC4103556

[B19] Inaba S. Tanabe K. Eda S. Ikeda S. Higashitani A. Mitsui H. (2009). Nitrous oxide emission and microbial community in the rhizosphere of nodulated soybeans during the late growth period. *Microbes Environ.* 24 64–67. 10.1264/jsme2.me08544 21566356

[B20] Intergovernmental Panel on Climate Change [IPCC] (2014). *Climate change 2014: Synthesis Report. Contribution of Working Groups I, II and III to the Fifth Assessment Report of the Intergovernmental Panel on Climate Change.* Geneva: IPCC.

[B21] Itakura M. Tabata K. Eda S. Mitsui H. Murakami K. Yasuda J. (2008). Generation of *Bradyrhizobium japonicum* mutants with increased N2O reductase activity by selection after introduction of a mutated dnaQ gene. *Appl. Environ. Microbiol.* 74 7258–7264. 10.1128/AEM.01850-08 18849448 PMC2592905

[B22] Itakura M. Uchida Y. Akiyama H. Takada-Hoshino Y. Shimomura Y. Morimoto S. (2013). Mitigation of nitrous oxide emissions from soils by *Bradyrhizobium japonicum* inoculation. *Nat. Clim. Chan.* 3 208–212. 10.1038/nclimate1734

[B23] Koropatkin N. M. Pakrasi H. B. Smith T. J. (2006). Atomic structure of a nitrate-binding protein crucial for photosynthetic productivity. *Proc. Natl. Acad. Sci. U.S.A.* 103 9820–9825. 10.1073/pnas.0602517103 16777960 PMC1502537

[B24] LeBauer D. S. Treseder K. (2008). Nitrogen limitation of net primary productivity in terrestrial ecosystems is globally distributed. *Ecology* 89 371–379. 10.1890/06-2057.1 18409427

[B25] Luque-Almagro V. M. Lyall V. J. Ferguson S. J. Roldán M. D. Richardson D. J. (2013). Nitrogen oxyanion-dependent dissociation of a two-component complex that regulates bacterial nitrate assimilation. *J. Biol. Chem.* 288 29692–29702. 10.1074/jbc.M113.459032 24005668 PMC3795266

[B26] Ohta H. Hattori T. (1983). Agromonas oligotrophica gen. nov., sp. nov., a nitrogen-fixing oligotrophic bacterium. *Antonie Van Leeuwenhoek* 49 429–446. 6651288 10.1007/BF00399322

[B27] Okubo T. Fukushima S. Itakura M. Oshima K. Longtonglang A. Teaumroong N. (2013). Genome analysis suggests that the soil oligotrophic bacterium *Agromonas oligotrophica* (*Bradyrhizobium oligotrophicum*) is a nitrogen-fixing symbiont of *Aeschynomene indica*. *Appl. Environ. Microbiol.* 79 2541–2551. 10.1128/AEM.00009-13 23396330 PMC3623176

[B28] Richardson D. J. Felgate H. Watmough N. Thomson A. Baggs E. (2009). Mitigating release of the potent greenhouse gas N2O from the nitrogen cycle-could enzymatic regulation hold the key? *Trends Biotechnol.* 27 388–397. 10.1016/j.tibtech.2009.03.009 19497629

[B29] Romeo A. Sonnleitner E. Sorger-Domenigg T. Nakano M. Eisenhaber B. Bläsi U. (2012). Transcriptional regulation of nitrate assimilation in *Pseudomonas aeruginosa* occurs via transcriptional antitermination within the nirBD-PA1779-cobA operon. *Microbiology* 158 1543–1552. 10.1099/mic.0.053850-0 22493305

[B30] Saeki Y. Nakamura M. Mason M. L. T. Yano T. Shiro S. Sameshima-Saito R. (2017). Effect of flooding and the nosZ gene in bradyrhizobia on bradyrhizobial community structure in the soil. *Microbes Environ.* 32 154–163. 10.1264/jsme2.ME16132 28592720 PMC5478539

[B31] Sameshima-Saito R. Chiba K. Hirayama J. Itakura M. Mitsui H. Eda S. (2006a). Symbiotic *Bradyrhizobium japonicum* reduces N2O surrounding the soybean root system via nitrous oxide reductase. *Appl. Environ. Microbiol.* 72 2526–2532. 10.1128/aem.72.4.2526-2532.2006 16597953 PMC1449076

[B32] Sameshima-Saito R. Chiba K. Minamisawa K. (2006b). Correlation of denitrifying capability with the existence of nap, nir, nor and nos genes in diverse strains of soybean bradyrhizobia. *Microbes Environ.* 21 174–184. 10.1264/jsme2.21.174

[B33] Sameshima-Saito R. Chiba K. Minamisawa K. (2004). New method of denitrification analysis of *Bradyrhizobium* field isolates by gas chromatographic determination of 15N-labeled N2. *Appl. Environ. Microbiol.* 70 2886–2891. 10.1128/aem.70.5.2886-2891.2004 15128547 PMC404451

[B34] Sánchez C. Bedmar E. J. Delgado M. J. (2011). “Denitrification in legume-associated endosymbiotic bacteria,” in *Nitrogen Cycling in Bacteria: Molecular Analysis*, ed. Moir J. W. B. (Norfolk: Caister Academic Press), 197–210.

[B35] Sánchez C. Itakura M. Mitsui H. Minamisawa K. (2013). Linked expressions of nap and nos genes in a *Bradyrhizobium japonicum* mutant with increased N2O reductase activity. *Appl. Environ. Microbiol.* 79 4178–4180. 10.1128/AEM.00703-13 23624475 PMC3697546

[B36] Sánchez C. Itakura M. Okubo T. Matsumoto T. Yoshikawa H. Gotoh A. (2014). The nitrate-sensing NasST system regulates nitrous oxide reductase and periplasmic nitrate reductase in *Bradyrhizobium japonicum*. *Environ. Microbiol.* 16 3263–3274. 10.1111/1462-2920.12546 24947409

[B37] Sánchez C. Minamisawa K. (2018). Redundant roles of *Bradyrhizobium oligotrophicum* Cu-type (NirK) and cd_1_-type (NirS) nitrite reductase genes under denitrifying conditions. *FEMS Microbiol. Lett.* 365:fny015 10.1093/femsle/fny015 29361081

[B38] Sánchez C. Mitsui H. Minamisawa K. (2017). Regulation of nitrous oxide reductase genes by NasT-mediated transcription antitermination in *Bradyrhizobium diazoefficiens*. *Environ. Microbiol. Rep.* 9 389–396. 10.1111/1758-2229.12543 28474433

[B39] Sánchez C. Siqueira A. F. Mitsui H. Minamisawa K. (2019). Identification of genes regulated by the antitermination factor NasT during denitrification in *Bradyrhizobium diazoefficiens*. *Microbes Environ.* 10.1111/1758-2229.12543 [Epub ahead of print]. 31257307 PMC6759348

[B40] Shiina Y. Itakura M. Choi H. Seaki Y. Hayatsu M. Minamisawa K. (2014). Relationship between soil type and N2O reductase genotype (nosZ) of indigenous soybean bradyrhizobia: nosZ-minus populations are dominant in Andosols. *Microbes Environ.* 29 420–426. 10.1264/jsme2.ME14130 25476067 PMC4262367

[B41] Shu C. Y. J. Zhulin I. B. (2002). ANTAR: an RNA-binding domain in transcription antitermination regulatory proteins. *Trends Biochem. Sci.* 27 3–5. 10.1016/s0968-0004(01)02036-9 11796212

[B42] Siqueira A. F. Minamisawa K. Sánchez C. (2017). Anaerobic reduction of nitrate to nitrous oxide is lower in *Bradyrhizobium japonicum* than in *Bradyrhizobium diazoefficiens*. *Microbes Environ.* 32 398–401. 10.1264/jsme2.me17081 29109361 PMC5745027

[B43] Sullivan M. J. Gates A. J. Appia-Ayme C. Rowley G. Richardson D. J. (2013). Copper control of bacterial nitrous oxide emission and its impact on vitamin B12-dependent metabolism. *Proc. Natl. Acad. Sci. U.S.A.* 110 19926–19931. 10.1073/pnas.1314529110 24248380 PMC3856849

[B44] Thomson A. J. Giannopoulos G. Pretty J. Baggs E. M. Richardson D. J. (2012). Biological sources and sinks of nitrous oxide and strategies to mitigate emissions. *Phil. Trans. R. Soc. B.* 367 1157–1168. 10.1098/rstb.2011.0415 22451101 PMC3306631

[B45] Torres M. J. Bueno E. Jiménez-Leiva A. Cabrera J. J. Bedmar E. J. Mesa S. (2017). FixK2 is the main transcriptional activator of *Bradyrhizobium diazoefficiens* nosRZDYFLX genes in response to low oxygen. *Front. Microbiol.* 8:1621. 10.3389/fmicb.2017.01621 28912756 PMC5582078

[B46] Torres M. J. Simon J. Rowley G. Bedmar E. J. Richardson D. J. Gates A. J. (2016). Nitrous oxide metabolism in nitrate-reducing bacteria: physiology and regulatory mechanisms. *Adv. Microb. Physiol.* 68 353–432. 10.1016/bs.ampbs.2016.02.007 27134026

[B47] Tortosa G. Hidalgo A. Salas A. Bedmar E. J. Mesa S. Delgado M. J. (2016). Nitrate and flooding induce N2O emissions from soybean nodules. *Symbiosis* 67 125–133. 10.1007/s13199-015-0341-3

[B48] Uchida Y. Akiyama H. (2013). Mitigation of postharvest nitrous oxide emissions from soybean ecosystems: a review. *Soil Sci. Plant Nutr.* 59 477–487. 10.1080/00380768.2013.805433

[B49] Velasco L. Mesa S. Xu C. A. Delgado M. J. Bedmar E. J. (2004). Molecular characterization of nosRZDFYLX genes coding for denitrifying nitrous oxide reductase of *Bradyrhizobium japonicum*. *Antonie Van Leeuwenhoek* 85 229–235. 10.1023/b:anto.0000020156.42470.db 15028871

[B50] Wang B. Pierson L. S. III Rensing C. Gunatilaka M. K. Kennedy C. (2012). NasT-mediated antitermination plays an essential role in the regulation of the assimilatory nitrate reductase operon in *Azotobacter vinelandii*. *Appl. Environ. Microbiol.* 78 6558–6567. 10.1128/AEM.01720-12 22773651 PMC3426680

[B51] Yang L. Cai Z. (2005). The effect of growing soybean (*Glycine max*. L.) on N2O emission from soil. *Soil Biol. Biochem.* 37 1205–1209. 10.1016/j.soilbio.2004.08.027 16825470

[B52] Zumft W. G. (1997). Cell biology and molecular basis of denitrification. *Microbiol. Mol. Biol. R.* 61 533–616. 10.1128/mmbr.61.4.533-616.1997 PMC232623 9409151

[B53] Zumft W. G. Kroneck P. M. (2007). Respiratory transformation of nitrous oxide (N2O) to dinitrogen by bacteria and archaea. *Adv. Microb. Physiol.* 52 107–227. 10.1016/s0065-2911(06)52003-x 17027372

